# Supplementary material for: Comparative genomic analysis of the principal Cryptosporidium species that infect humans
Source: PeerJ. 2020 Dec 2;8:e10478. doi: 10.7717/peerj.10478 (PMC7718795; doi:10.7717/peerj.10478)
Supplement: Supplemental Information 1 [file peerj-08-10478-s001.docx]

**Table 1.**

**Selected Genomes for comparative analysis.**

| **Species** | **Genomes ID** | **gp60 Subtype** | **Geographic sources** | **Isolation source** | **Propagated in animals** | **Sequencing techniques** | **BioProject ID** | **References** |
| --- | --- | --- | --- | --- | --- | --- | --- | --- |
| *C. parvum* | UKP2 | IIaA19G1R2 | UK: England | Human feces | N/A | Illumina MiSeq | PRJNA253836 | (Hadfield et al., 2015) |
|  | UKP3 | IIaA18G2R1 | UK: Wales | Human feces | N/A | Illumina HiSeq | PRJNA253840 | (Hadfield et al., 2015) |
|  | UKP4 | IIaA15G2R1 | UK: England | Human feces | N/A | Illumina HiSeq | PRJNA253843 | (Hadfield et al., 2015) |
|  | UKP5 | IIaA15G2R1 | UK | Human feces | N/A | Illumina HiSeq | PRJNA253845 | (Hadfield et al., 2015) |
|  | UKP6 | IIaA15G2R1 | UK | Human feces | N/A | Illumina HiSeq | PRJNA253846 | (Hadfield et al., 2015) |
|  | UKP7 | IIaA17G1R1 | UK: England | Human feces | N/A | Illumina HiSeq | PRJNA253847 | (Hadfield et al., 2015) |
|  | UKP8 | IIdA22G1 | UK: England | Human feces | N/A | Illumina HiSeq | PRJNA253848 | (Hadfield et al., 2015) |
|  | UKP14 | IIcA5G3a | UK: England | Human feces | N/A | Illumina HiSeq | PRJNA315506 | (Nader et al., 2019) |
|  | UKP15 | IIcA5G3a | UK: Wales | Human feces | N/A | Illumina HiSeq | PRJNA315507 | (Nader et al., 2019) |
| *C. hominis* | UKH1 | IbA10G2 | UK: Wales | Human feces | Gnotobiotic piglets | Illumina MiSeq | PRJNA222837 | (Ifeonu et al., 2016) |
|  | UKH3 | IbA10G2 | UK: England | Human feces | N/A | Illumina MiSeq | PRJNA253834 | (Hadfield et al., 2015) |
|  | UKH4 | IaA14R3 | UK: England | Human feces | N/A | Illumina HiSeq | PRJNA253838 | (Hadfield et al., 2015) |
|  | UKH5 | IbA10G2 | UK: England | Human feces | N/A | Illumina HiSeq | PRJNA253839 | (Hadfield et al., 2015) |
|  | 37999 | IbA10G2 | USA: Twin Fall, Idaho | Human feces | N/A | Illumina GAIIx | PRJNA252787 | (Guo et al., 2015a) |
|  | TU502_2012 | IbA10G2 | Uganda | Human feces | Gnotobiotic piglets | Illumina MiSeq | PRJNA222836 | (Ifeonu et al., 2016) |
|  | 30976 | IaA28R4 | USA: St. Louis | Human feces | N/A | Illumina GAIIx | PRJNA252787 | (Guo et al., 2015a) |
|  | UdeA01 | IeA11G3T3 | Colombia: Medellín | Human feces | N/A | Illumina MiSeq | PRJEB10000 | (Isaza et al., 2015) |
|  | SWEH2 | IdA14 | Kenya or Rwanda | Human feces | N/A | Ion Torrent | PRJNA307563 | (Sikora, Arrighi, Beser, & Andersson, 2017) |
|  | SWEH5 | IfA12G1 | Denmark | Human feces | N/A | Ion Torrent | PRJNA307563 | (Sikora, Arrighi, et al., 2017) |
| *C. meleagridis* | UKMEL1 | IIIbA22G1R1c | India | Human feces | Mice | Illumina MiSeq | PRJNA222838 | (Ifeonu et al., 2016) |
|  | UKMEL3 | IIIgA23G3 | UK | Human feces | N/A | Illumina HiSeq | PRJNA315502 | (Nader et al., 2019) |
|  | UKMEL4 | IIIhA7 | UK | Human feces | N/A | Illumina HiSeq | PRJNA315503 | (Nader et al., 2019) |
|  | TU1867 | IIIeA21G2R1 | Uganda | Human feces | Gnotobiotic piglets, mice, and chickens | Illumina MiSeq | PRJNA192428 | (Akiyoshi et al., 2003) |
