# Supplementary material for: Comparative genomic analysis of the principal Cryptosporidium species that infect humans
Source: PeerJ. 2020 Dec 2;8:e10478. doi: 10.7717/peerj.10478 (PMC7718795; doi:10.7717/peerj.10478)
Supplement: Supplemental Information 2 — + Assemblies retrieved from CryptoDB database release 43. * de novo assemblies filtered by Bit score value ≥ 300. aReads were mapped using BWA to the reference C. parvum Iowa II genome retrieved from CryptoDB database release 43. b Reads were mapped using BWA to the de novo assembled contigs. [file peerj-08-10478-s002.docx]

**Table 2.**

**Summary of genome assembly data of *Cryptosporidium* isolates.**

+ Assemblies retrieved from CryptoDB database release 43.

* *de novo* assemblies filtered by Bit score value ≥ 300.

^a^Reads were mapped using BWA to the reference *C. parvum* Iowa II genome retrieved from CryptoDB database release 43.

^b^Reads were mapped using BWA to the *de novo* assembled contigs.

| **Species** | **Genome ID** | **No. of reads** | **Assembly size (bp)** | **No. of contigs** | **Largest contig (bp)** | **Contig N_50_ (bp)** | **G + C content (%)** | **No. of Ns** | **a. Mean coverage**  **C. parvum Iowa II genome (X)** | **b. Mean coverage** ***de novo* assembled contigs (X)** |
| --- | --- | --- | --- | --- | --- | --- | --- | --- | --- | --- |
| *C. parvum* | UKP2 * | 2,015,213 | 9,113,527 | 118 | 859,882 | 216,383 | 30.17 | 485 | 52.3 | 52.4 |
|  | UKP3 * | 8,227,141 | 9,636,677 | 2971 | 364,354 | 51,320 | 30.87 | 25,249 | 242.4 | 229.3 |
|  | UKP4 * | 9,342,641 | 9,429,553 | 1933 | 418,794 | 62,049 | 30,59 | 18,349 | 279.5 | 270.2 |
|  | UKP5 * | 1,321,400 | 9,017,288 | 473 | 376,426 | 68,305 | 30.24 | 254 | 40.1 | 40.4 |
|  | UKP6 * | 5,598,532 | 9,129,374 | 64 | 1,336,351 | 383,563 | 30.18 | 1,484 | 160.3 | 160.4 |
|  | UKP7 * | 3,746,781 | 9,007,490 | 555 | 340,451 | 67,504 | 30.32 | 861 | 112.3 | 113.8 |
|  | UKP8 * | 11,161,344 | 9,108,067 | 451 | 787,834 | 95,411 | 30.24 | 2,482 | 300.4 | 301.5 |
|  | UKP14 * | 2,378,388 | 8,221,521 | 2787 | 43,566 | 5,120 | 31.95 | 441 | 69.4 | 85.4 |
|  | UKP15 * | 6,627,377 | 8,904,901 | 1830 | 94,121 | 10,293 | 31.09 | 693 | 147.6 | 209.8 |
| *C. hominis* | UKH1 + | 3,798,205 | 9,141,398 | 156 | 542,781 | 179,408 | 30.13 | 0 | 191.7 | 197.3 |
|  | UKH3 * | 1,238,762 | 9,071,292 | 179 | 552,915 | 167,737 | 30.14 | 256 | 35.2 | 35.8 |
|  | UKH4 * | 11,895,367 | 9,390,791 | 2164 | 239,037 | 48,766 | 30.66 | 12,842 | 326.2 | 321.5 |
|  | UKH5 * | 12,649,912 | 9,068,487 | 526 | 730,267 | 81,885 | 30.20 | 2,681 | 354.8 | 362.2 |
|  | 30976 + | 35,360,353 | 9,059,225 | 53 | 1,279,890 | 470,636 | 30.12 | 1,699 | 502.4 | 511.0 |
|  | 37999 + | 16,569,871 | 9,054,010 | 78 | 1,029,232 | 406,678 | 30.13 | 3,168 | 349.9 | 367.4 |
|  | TU502-2012 + | 1,810,060 | 9,107,739 | 119 | 1,270,815 | 238,509 | 30.13 | 0 | 94,8 | 96.0 |
|  | UdeA01 + | 1,080,448 | 9,043,938 | 8 | 1,322,947 | 1,103,974 | 30.15 | 8,500 | 52.6 | 53.4 |
|  | SWEH2 * | 1,791,829 | 8,818,287 | 1629 | 62,020 | 9,465 | 30.43 | 0 | 33.9 | 35.2 |
|  | SWEH5 * | 2,058,197 | 8,821,738 | 1342 | 94,133 | 14,514 | 30.47 | 96 | 40.8 | 42.4 |
| *C. meleagridis* | UKMEL1 + | 11,431,022 | 8,973,200 | 57 | 732,862 | 322,908 | 30.97 | 0 | 101.6 | 110.4 |
|  | UKMEL3 * | 3,097,090 | 9,028,762 | 421 | 214,804 | 62,491 | 31.01 | 6,697 | 75.2 | 93.1 |
|  | UKMEL4 * | 5,648,885 | 9,150,400 | 167 | 296,518 | 110,331 | 30.94 | 693 | 148.7 | 182.4 |
|  | TU1867 * | 16,007,104 | 8,997,078 | 46 | 1,076,730 | 672,302 | 30.94 | 763 | 344.7 | 378.5 |
