# Supplementary material for: Comparative genomic analysis of the principal Cryptosporidium species that infect humans
Source: PeerJ. 2020 Dec 2;8:e10478. doi: 10.7717/peerj.10478 (PMC7718795; doi:10.7717/peerj.10478)
Supplement: Supplemental Information 3 — The contigs from de novo assembly for each genome were aligned with reference C. parvum Iowa II genome retrieved from CryptoDB database release 43. [file peerj-08-10478-s003.docx]

**Table 3.**

**Alignment results of *Cryptosporidium* genomes against reference *C. parvum* Iowa II genome.**

The contigs from *de novo* assembly for each genome were aligned with reference *C. parvum* Iowa II genome retrieved from CryptoDB database release 43.

| **Species** | **Genome ID** | **Similarity to Iowa II (%)** | **Aligned bases (%)** | **Total SNVs** | **SNVs in the genome compared to *C. parvum* (%)** |
| --- | --- | --- | --- | --- | --- |
| *C. parvum* | UKP2 | 99.93 | 99.20 | 1,595 | 0.02 |
|  | UKP3 | 99.91 | 99.25 | 1,978 | 0.02 |
|  | UKP4 | 99.92 | 99.32 | 1,930 | 0.02 |
|  | UKP5 | 99.91 | 99.51 | 2,674 | 0.03 |
|  | UKP6 | 99.93 | 99.39 | 1,612 | 0.02 |
|  | UKP7 | 99.86 | 99.61 | 2,116 | 0.02 |
|  | UKP8 | 99.77 | 99.47 | 5,752 | 0.06 |
|  | UKP14 | 99.58 | 96.91 | 18,471 | 0.22 |
|  | UKP15 | 99.51 | 97.38 | 19,923 | 0.22 |
| *C. hominis* | 30976 | 96.82 | 99.00 | 223,712 | 2.47 |
|  | UdeA01 | 96.84 | 99.29 | 223,031 | 2.47 |
|  | 37999 | 96.81 | 99.23 | 223,568 | 2.47 |
|  | TU502_2012 | 96.81 | 99.22 | 224,430 | 2.46 |
|  | UKH1 | 96.81 | 99.22 | 223,901 | 2.45 |
|  | UKH3 | 96.81 | 99.26 | 224,460 | 2.47 |
|  | UKH4 | 96.87 | 98.02 | 221,714 | 2.36 |
|  | UKH5 | 96.84 | 99.20 | 223,819 | 2.47 |
|  | SWEH2 | 96.88 | 99.33 | 213,206 | 2.42 |
|  | SWEH5 | 96.85 | 99.38 | 215,136 | 2.44 |
| *C. meleagridis* | UKMEL1 | 91.55 | 97.40 | 624,528 | 6.96 |
|  | UKMEL3 | 91.59 | 96.96 | 619,036 | 6.86 |
|  | UKMEL4 | 91.54 | 97.27 | 624,685 | 6.83 |
|  | TU1867 | 91.53 | 97.34 | 625,265 | 6.95 |
