# Supplementary material for: Comparative genomic analysis of the principal Cryptosporidium species that infect humans
Source: PeerJ. 2020 Dec 2;8:e10478. doi: 10.7717/peerj.10478 (PMC7718795; doi:10.7717/peerj.10478)
Supplement: Supplemental Information 5 — * Unique gene in C. meleagridis that presented two deletions at different coordinates. † Largest insertion detected in the genomes of this species. ±Deletions shared by all genomes of C. meleagridis and C. hominis. [file peerj-08-10478-s005.docx]

**Table 5. Annotation of genes affected by indel events**

| ***C. meleagridis* indels in 100% of genomes (4 of 4)** | | | | |
| --- | --- | --- | --- | --- |
| **Orthologous gene in *C. parvum* Iowa** | **Chromosome** | **CryptoDB Version 46 Annotation** | **Indel** | **Length (bp)** |
| **cgd2_540** | 2 | Uncharacterized Protein | Deletion | 132 |
| **cgd2_690 *** | 2 | Uncharacterized Protein | 2 Deletions | 70 y 93 |
| **cgd3_1540** | 3 | Signal peptide containing protein | Deletion | 78 |
| **cgd4_200** | 4 | Uncharacterized Protein | Deletion | 73 |
| **cgd4_770** | 4 | Uncharacterized Protein | Deletion | 148 |
| **cgd4_1010** | 4 | Galactose-binding domain-containing protein | Deletion | 108 |
| **cgd5_220** | 5 | Uncharacterized Protein | Deletion | 96 |
| **cgd5_550** | 5 | Integrator complex subunit 9-like protein | Deletion | 54 |
| **cgd5_1940** | 5 | Uncharacterized Protein | Insertion | 278 |
| **cgd5_2180 †** | 5 | Uncharacterized Protein | Insertion | 1035 |
| **cgd5_2250** | 5 | Uncharacterized Protein | Deletion | 114 |
| **cgd5_4490** | 5 | Uncharacterized Protein | Deletion | 54 |
| **cgd6_830** | 6 | Uncharacterized Protein | Insertion | 105 |
| **cgd6_4290 ±** | 6 | MATH/TRAF domain/WD40 repeat containing protein | Deletion | 99 |
| **cgd6_5400** | 6 | Uncharacterized Protein | Deletion | 59 |
| **cgd7_420 ±** | 7 | Protein with DEXDc ring HELICc possible SNF2 domain | Deletion | 144 |
| **cgd7_890** | 7 | WD40/YVTN repeat-like-containing protein | Deletion | 108 |
| **cgd7_1300** | 7 | Protein kinase-like domain-containing protein | Deletion | 123 |
| **cgd7_1310** | 7 | Glycosyltransferase 2-like protein | Deletion | 259 |
| **cgd7_1330** | 7 | Serine/Threonine protein kinase | Deletion | 96 |
| **cgd7_4020** | 7 | Cryptopsoridial mucin | Deletion | 79 |
| **cgd8_410** | 8 | Uncharacterized Protein | Deletion | 72 |
| **cgd8_680** | 8 | Uncharacterized Protein | Deletion | 120 |
| **cgd8_1570** | 8 | CCCH like finger domain nucleoporin | Deletion | 171 |
| **cgd8_2040** | 8 | Potassium channel tetramerisation-type BTB domain | Deletion | 120 |
| **cgd8_2770** | 8 | Uncharacterized Protein | Deletion | 73 |
| **cgd8_5190** | 8 | Uncharacterized Protein | Deletion | 225 |
| ***C. hominis* indels in 100% of genomes (10 of 10)** | | | | |
| **cgd6_4290** | 6 | Deleción MATH/TRAF domain/WD40 repeat-containing protein | Deletion | 99 |
| **cgd7_420** | 7 | Protein with DEXDc ring HELICc possible SNF2 domain | Deletion | 144 |
| **cgd7_500** | 7 | RTR1-type zinc finger | Insertion | 54 |
| ***C. parvum* indels in 56% of genomes (5 of 9)** | | | | |
| **cgd3_190** | 3 | Fibrillin/EGF-like calcium-binding conserved site-containing protein | Insertion | 100 |
| **cgd3_3900** | 3 | Uncharacterized Protein | Insertion | 100 |

* Unique gene in *C. meleagridis* that presented two deletions at different coordinates. † Largest insertion detected in the genomes of this species. ± Deletions shared by all genomes of *C. meleagridis* and *C. hominis*.
